# Changes in HIV Preexposure Prophylaxis Awareness and Use Among Men Who Have Sex with Men — 20 Urban Areas, 2014 and 2017

**DOI:** 10.15585/mmwr.mm6827a1

**Published:** 2019-07-12

**Authors:** Teresa Finlayson, Susan Cha, Ming Xia, Lindsay Trujillo, Damian Denson, Joseph Prejean, Dafna Kanny, Cyprian Wejnert, National HIV Behavioral Surveillance Study Group, National HIV Behavioral Surveillance Study Group, Meaghan Abrego, Alia Al-Tayyib, Bridget Anderson, Narquis Barak, Lissa Bayang, Jeremy M. Beckford, Nanette Benbow, Barbara Bolden, Kathleen A. Brady, Mary-Grace Brandt, Sarah Braunstein, Richard Burt, Rosalinda Cano, Sidney Carrillo, Jie Deng, Rose Doherty, Anna Flynn, Colin Flynn, David Forrest, Dawn Fukuda, Danielle German, Sara Glick, Henry Godette, Vivian Griffin, Emily Higgins, Theresa Ick, Tom Jaenicke, Antonio D. Jimenez, Salma Khuwaja, Monina Klevens, Irene Kuo, Marlene LaLota, Zaida Lopez, Yingbo Ma, Kathryn Macomber, Stephanie Masiello Schuette, Melanie Mattson, David Melton, Sandra Miranda De León, Alan Neaigus, Willie Nixon, Chrysanthus Nnumolu, Alicia Novoa, Conall O’Cleirigh, Jenevieve Opoku, Paige Padgett, Jonathon Poe, Nikhil Prachand, H. Fisher Raymond, Hafeez Rehman, Kathleen H. Reilly, Alexis Rivera, William T. Robinson, Yadira Rolón-Colón, Kimi Sato, John-Mark Schacht, Ekow Kwa Sey, Shane Sheu, Jennifer Shinefeld, Mark Shpaner, Amber Sinclair, Lou Smith, Emma Spencer, Ashley Tate, Hanne Thiede, Jeff Todd, Veronica Tovar-Moore, Margaret Vaaler, Chris Wittke, Afework Wogayehu, Pascale Wortley, Meagan C. Zarwell

**Affiliations:** ^1^Division of HIV/AIDS Prevention, National Center for HIV/AIDS, Viral Hepatitis, STD, and TB Prevention, CDC; ^2^ICF International, Fairfax, Virginia; ^3^Oak Ridge Institute for Science and Education, Oak Ridge, Tennessee.; Nassau and Suffolk counties, New York; Denver, Colorado; Nassau and Suffolk counties, New York; New Orleans, Louisiana; San Diego, California; New Orleans, Louisiana; Chicago, Illinois; Newark, New Jersey; Philadelphia, Pennsylvania; Detroit, Michigan; New York City, New York; Seattle, Washington; San Diego, California; New York City, New York; Dallas, Texas; Boston, Massachusetts; San Diego, California; Baltimore, Maryland; Miami, Florida; Boston, Massachusetts; Baltimore, Maryland; Seattle, Washington; Newark, New Jersey; Detroit, Michigan; Detroit, Michigan; San Francisco, California; Seattle, Washington; Chicago, Illinois; Houston, Texas; Boston, Massachusetts; Washington, D.C; Miami, Florida; Houston, Texas; Los Angeles, California; Detroit, Michigan; Chicago, Illinois; Denver, Colorado; Atlanta, Georgia; San Juan, Puerto Rico; New York City, New York; Miami, Florida; Philadelphia, Pennsylvania; Dallas, Texas; Boston, Massachusetts; Washington, D.C; Houston, Texas; Dallas, Texas; Chicago, Illinois; San Francisco, California; Houston, Texas; New York City, New York; New York City, New York; New Orleans, Louisiana; San Juan, Puerto Rico; Atlanta, Georgia; Miami, Florida; Los Angeles, California; Dallas, Texas; Philadelphia, Pennsylvania; Philadelphia, Pennsylvania; Nassau and Suffolk counties, New York; Nassau and Suffolk counties, New York; Miami, Florida; Nassau and Suffolk counties, New York; Seattle, Washington; Atlanta, Georgia; San Diego, California; Dallas, Texas; Boston, Massachusetts; Newark, New Jersey; Atlanta, Georgia; New Orleans, Louisiana

In February 2019, the U.S. Department of Health and Human Services proposed a strategic initiative to end the human immunodeficiency (HIV) epidemic in the United States by reducing new HIV infections by 90% during 2020–2030* (*1*). Phase 1 of the Ending the HIV Epidemic initiative focuses on Washington, DC; San Juan, Puerto Rico; and 48 counties where the majority of new diagnoses of HIV infection in 2016 and 2017 were concentrated and on seven states with a disproportionate occurrence of HIV in rural areas relative to other states.^†^ One of the four pillars in the initiative is protecting persons at risk for HIV infection using proven, comprehensive prevention approaches and treatments, such as HIV preexposure prophylaxis (PrEP), which is the use of antiretroviral medications that have proven effective at preventing infection among persons at risk for acquiring HIV. In 2014, CDC released clinical PrEP guidelines to health care providers (*2*) and intensified efforts to raise awareness and increase the use of PrEP among persons at risk for infection, including gay, bisexual, and other men who have sex with men (MSM), a group that accounted for an estimated 68% of new HIV infections in 2016 (*3*). Data from CDC’s National HIV Behavioral Surveillance (NHBS) were collected in 20 U.S. urban areas in 2014 and 2017, covering 26 of the geographic areas included in Phase I of the Ending the HIV Epidemic initiative, and were compared to assess changes in PrEP awareness and use among MSM. From 2014 to 2017, PrEP awareness increased by 50% overall, with >80% of MSM in 17 of the 20 urban areas reporting PrEP awareness in 2017. Among MSM with likely indications for PrEP (e.g., sexual risk behaviors or recent bacterial sexually transmitted infection [STI]), use of PrEP increased by approximately 500% from 6% to 35%, with significant increases observed in all urban areas and in almost all demographic subgroups. Despite this progress, PrEP use among MSM, especially among black and Hispanic MSM, remains low. Continued efforts to improve coverage are needed to reach the goal of 90% reduction in HIV incidence by 2030. In addition to developing new ways of connecting black and Hispanic MSM to health care providers through demonstration projects, CDC has developed resources and tools such as the Prescribe HIV Prevention program to enable health care providers to integrate PrEP into their clinical care.^§^ By routinely testing their patients for HIV, assessing HIV-negative patients for risk behaviors, and prescribing PrEP as needed, health care providers can play a critical role in this effort.

NHBS staff members in 20 urban areas collected cross-sectional behavioral survey data and conducted HIV testing among MSM at recruitment events using venue-based sampling^¶^ (*4*). Eligible participants** completed a standardized questionnaire administered in person by trained interviewers. All participants were offered anonymous HIV testing and incentives for the interview and HIV test.^††^ Analysis was limited to eligible participants at risk for HIV infection who were likely to meet clinical indications for PrEP^§§^ (*2*). Specifically, the analysis was limited to MSM who had a negative NHBS HIV test result, did not report a previous HIV-positive test result, had either one male sex partner who was HIV-positive or two or more male sex partners in the past 12 months, and reported either condomless anal sex or a bacterial STI (i.e., syphilis, gonorrhea, or chlamydia) in the past 12 months. PrEP awareness and use were measured differently in 2014 and in 2017. In 2014, participants were asked whether they had “ever heard of people who do not have HIV taking anti-HIV medicines, to keep from getting HIV” and whether, in the past 12 months, they had “taken anti-HIV medicines before sex because you thought it would keep you from getting HIV.” In 2017, participants were informed that PrEP is an antiretroviral medicine taken for months or years by a person who is HIV-negative to reduce the risk for getting HIV and then asked whether they had ever heard of PrEP and whether, in the past 12 months they had taken PrEP to reduce the risk of getting HIV. Log-linked Poisson regression models with generalized estimating equations clustered on recruitment event were stratified by subgroup to estimate prevalence ratios and 95% confidence intervals (CIs) for PrEP awareness and use by year. Stratified models for each subgroup were adjusted for income, health insurance, and region. Analyses were conducted using SAS software (version 9.4; SAS Institute).

In 2014 and 2017, 18,610 sexually active MSM were interviewed (9,640 in 2014; 8,970 in 2017) in the 20 urban areas. Of those, this analysis is limited to 7,873 MSM (42%) who had a negative HIV test result but were at risk for HIV infection and likely met the clinical indications for PrEP (3,821 [40%] in 2014; 4,052 [45%] in 2017). From 2014 to 2017, awareness of PrEP among these MSM increased overall from 60% to 90% (adjusted prevalence ratio [aPR] = 1.45; 95% CI = 1.41–1.50) and increased in all urban areas and subgroups (Table 1). In 2017, >80% of MSM in 17 of 20 urban areas and in most demographic subgroups were aware of PrEP. From 2014 to 2017, use of PrEP among MSM increased overall from 6% to 35% (aPR = 5.66; 95% CI = 4.85–6.61) and increased in all urban areas and in almost all demographic subgroups (Table 2). Substantial increases in PrEP use occurred among black, Hispanic, and young (aged 18–29 years) MSM from 2014 to 2017. In 2017, the differences in PrEP use between Hispanic (30%) and white (42%) MSM (aPR = 0.91; 95% CI = 0.78–1.06) and between young (32%) and older (38%) MSM (aPR = 0.97; 95% CI = 0.89–1.05) were no longer significant after controlling for income, health insurance, and region. However, the difference in reported PrEP use between black (26%) and white (42%) MSM remained significant after controlling for these three factors (aPR = 0.78; 95% CI = 0.66–0.92). During 2017, PrEP use increased with education and income, and 39% of the MSM who saw a health care provider in the past 12 months reported PrEP use.

**TABLE 1:** Number and percentage of men who have sex with men who are at risk for human immunodeficiency virus (HIV) infection* and reported awareness of HIV preexposure prophylaxis, by demographic characteristics — National HIV Behavioral Surveillance System, United States, 2014 and 2017

Characteristic	2014		2017		Adjusted prevalence ratio^†^ (95% CI)
	No. (%)	Total	No. (%)	Total	
**Overall**	**2,286 (59.8)**	**3,821**	**3,664 (90.4)**	**4,052**	**1.45 (1.41–1.50)**
**Age group (yrs)**					
18–29	1,115 (57.5)	1,939	1,717 (91.2)	1,882	1.52 (1.45–1.59)
≥30	1,171 (62.2)	1,882	1,947 (89.7)	2,170	1.40 (1.34–1.46)
**Racial/Ethnic group**					
Black	376 (47.1)	798	729 (86.2)	846	1.76 (1.62–1.92)
Hispanic/Latino	529 (48.9)	1,081	1,032 (86.6)	1,191	1.66 (1.55–1.77)
White	1,152 (71.7)	1,607	1,555 (94.5)	1,645	1.30 (1.25–1.35)
Other^§^	216 (68.4)	316	322 (93.6)	344	1.36 (1.25–1.48)
**Sexual identity**					
Heterosexual	12 (38.7)	31	12 (60.0)	20	1.55 (0.87–2.76)
Homosexual or gay	2,038 (63.3)	3,222	3,126 (92.2)	3,389	1.41 (1.36–1.45)
Bisexual	227 (40.9)	555	513 (81.4)	630	1.90 (1.71–2.12)
**Education**					
High school degree or less	353 (38.8)	910	604 (80.5)	750	1.98 (1.79–2.17)
Some college or vocational school	695 (56.2)	1,237	1,184 (90.5)	1,309	1.56 (1.48–1.65)
College degree or graduate studies	1,237 (73.9)	1,673	1,875 (94.1)	1,992	1.26 (1.22–1.30)
**Household income**					
$0–$24,999	593 (45.5)	1,303	838 (82.2)	1,019	1.73 (1.61–1.85)
$25,000–$49,999	622 (61.5)	1,012	1,000 (91.0)	1,099	1.46 (1.38–1.55)
$50,000–$74,999	428 (66.9)	640	755 (93.8)	805	1.39 (1.31–1.48)
≥$75,000	620 (75.7)	819	1,058 (95.3)	1,110	1.26 (1.20–1.31)
**Currently have health insurance**					
No	463 (51.1)	906	621 (85.5)	726	1.59 (1.48–1.72)
Yes	1,818 (62.6)	2,906	3,039 (91.6)	3,319	1.42 (1.37–1.47)
**Visited a health care provider within the past 12 months**					
No	332 (47.1)	705	409 (78.8)	519	1.60 (1.46–1.76)
Yes	1,953 (62.7)	3,114	3,254 (92.1)	3,532	1.42 (1.37–1.47)
**Usual source of health care**					
No usual place for health care	386 (46.3)	834	570 (83.3)	684	1.72 (1.58–1.87)
Clinic or health care center	599 (61.8)	970	1,053 (91.3)	1,153	1.43 (1.35–1.51)
Doctor’s office or HMO	1,218 (64.8)	1,881	1,888 (92.6)	2,039	1.39 (1.34–1.44)
Other place for health care	57 (62.0)	92	115 (87.8)	131	1.42 (1.19–1.69)
**Participated in a behavioral Intervention within the past 12 months**					
No	1,627 (57.2)	2,842	2,486 (88.9)	2,797	1.49 (1.43–1.55)
Yes	659 (67.3)	979	1,176 (93.9)	1,253	1.33 (1.27–1.40)
**Tested for HIV within the past 12 months**					
No	348 (41.5)	838	452 (75.1)	602	1.73 (1.57–1.91)
Yes	1,935 (65.0)	2,976	3,207 (93.1)	3,444	1.39 (1.35–1.43)
**Region^¶^**					
Midwest	216 (61.2)	353	289 (80.7)	358	1.29 (1.13–1.46)
Northeast	471 (59.4)	793	718 (90.4)	794	1.51 (1.40–1.62)
South	755 (55.9)	1,350	1,239 (89.6)	1,383	1.53 (1.44–1.62)
U.S. territories	63 (27.6)	228	82 (66.7)	123	2.25 (1.75–2.89)
West	781 (71.2)	1,097	1,336 (95.8)	1,394	1.34 (1.28–1.41)
**Urban area**					
Atlanta, GA	119 (62.0)	192	184 (92.5)	199	1.43 (1.25–1.64)
Baltimore, MD	87 (55.4)	157	89 (82.4)	108	1.52 (1.28–1.81)
Boston, MA	106 (73.1)	145	203 (96.7)	210	1.33 (1.18–1.49)
Chicago, IL	162 (82.2)	197	186 (94.4)	197	1.13 (1.05–1.22)
Dallas, TX	59 (33.1)	178	224 (89.2)	251	2.28 (1.76–2.97)
Denver, CO	122 (58.1)	210	270 (93.8)	288	1.61 (1.41–1.83)
Detroit, MI	54 (34.6)	156	103 (64.0)	161	1.80 (1.41–2.31)
Houston, TX	93 (49.7)	187	212 (86.5)	245	1.67 (1.38–2.01)
Los Angeles, CA	177 (68.3)	259	287 (97.3)	295	1.44 (1.31–1.57)
Miami, FL	98 (46.4)	211	134 (78.8)	170	1.67 (1.40–2.00)
Nassau and Suffolk counties, NY	73 (45.9)	159	68 (84.0)	81	1.83 (1.50–2.23)
New Orleans, LA	100 (55.2)	181	156 (94.5)	165	1.66 (1.42–1.94)
New York City, NY	125 (80.1)	156	236 (95.2)	248	1.17 (1.08–1.27)
Newark, NJ	22 (25.0)	88	48 (88.9)	54	3.73 (2.69–5.18)
Philadelphia, PA	145 (59.2)	245	163 (81.1)	201	1.36 (1.18–1.57)
San Diego, CA	139 (63.8)	218	277 (94.2)	294	1.47 (1.30–1.67)
San Francisco, CA	158 (90.8)	174	261 (97.4)	268	1.05 (1.00–1.12)
San Juan, PR	63 (27.6)	228	82 (66.7)	123	2.25 (1.75–2.89)
Seattle, WA	185 (78.4)	236	241 (96.8)	249	1.24 (1.16–1.33)
Washington, DC	199 (81.6)	244	240 (98.0)	245	1.19 (1.12–1.27)

**Abbreviations:** CI = confidence interval; HMO = health maintenance organization.

* Men who were at risk for HIV infection and likely to meet clinical indications for HIV preexposure prophylaxis. This was defined as men who had a negative HIV test result at the time of the interview, did not report a previous HIV-positive test result, had either one male sex partner who was HIV-positive or multiple male sex partners in the past 12 months, and reported either condomless anal sex or a sexually transmitted bacterial infection in the past 12 months.

^†^ Models adjusted for income, health insurance, and region.

^§^ Includes American Indian, Alaskan Native, Asian, Native Hawaiian, Pacific Islander, or multiple races.

^¶^ *Midwest region* includes Chicago, IL and Detroit, MI. *Northeast* *region* includes Boston, MA; Nassau and Suffolk counties, NY; New York City, NY; Newark, NJ; and Philadelphia, PA. *South region* includes Atlanta, GA; Baltimore, MD; Dallas, TX; Houston, TX; Miami, FL; New Orleans, LA; and Washington, DC. *U.S. territories region* includes San Juan, PR. *West region* includes Denver, CO; Los Angeles, CA; San Diego, CA; San Francisco, CA; and Seattle, WA.

**TABLE 2:** Number and percentage of men who have sex with men who are at risk for human immunodeficiency virus (HIV) infection* and reported using HIV preexposure prophylaxis, by demographic characteristics — National HIV Behavioral Surveillance System, United States, 2014 and 2017

Characteristic	2014		2017		Adjusted prevalence ratio^†^ (95% CI)
	No. (%)	Total	No. (%)	Total	
**Overall**	**216 (5.7)**	**3,821**	**1,425 (35.1)**	**4,052**	**5.66 (4.85–6.61)**
**Age group (yrs)**					
18–29	90 (4.6)	1,939	608 (32.3)	1,882	6.36 (5.05–8.02)
≥30	126 (6.7)	1,882	817 (37.6)	2,170	5.21 (4.30–6.32)
**Racial/Ethnic group**					
Black	30 (3.8)	798	222 (26.2)	846	6.44 (4.36–9.51)
Hispanic/Latino	41 (3.8)	1,081	357 (30.0)	1,191	6.92 (5.08–9.44)
White	133 (8.3)	1,607	697 (42.4)	1,645	4.83 (3.96–5.88)
Other^§^	12 (3.8)	316	137 (39.8)	344	9.53 (5.36–16.96)
**Sexual identity**					
Heterosexual	2 (6.5)	31	3 (15.0)	20	2.33 (0.42–12.78)
Homosexual or gay	196 (6.1)	3,222	1,273 (37.6)	3,389	5.65 (4.81–6.63)
Bisexual	18 (3.2)	555	144 (22.9)	630	6.43 (3.96–10.45)
**Education**					
High school degree or less	19 (2.1)	910	192 (25.6)	750	10.76 (6.69–17.33)
Some college or vocational school	55 (4.4)	1,237	390 (29.8)	1,309	6.77 (5.14–8.92)
College degree or graduate studies	142 (8.5)	1,673	842 (42.3)	1,992	4.80 (3.99–5.77)
**Household income**					
$0–$24,999	48 (3.7)	1,303	264 (25.9)	1,019	6.20 (4.51–8.52)
$25,000–$49,999	45 (4.4)	1,012	346 (31.5)	1,099	6.82 (5.00–9.32)
$50,000–$74,999	34 (5.3)	640	294 (36.5)	805	6.89 (4.89–9.71)
≥$75,000	88 (10.7)	819	521 (46.9)	1,110	4.29 (3.43–5.37)
**Currently have health insurance**					
No	23 (2.5)	906	134 (18.5)	726	6.63 (4.35–10.10)
Yes	192 (6.6)	2,906	1,290 (38.9)	3,319	5.53 (4.70–6.51)
**Visited a health care provider within the past 12 months**					
No	5 (0.7)	705	37 (7.1)	519	9.81 (3.87–24.85)
Yes	211 (6.8)	3,114	1,388 (39.3)	3,532	5.38 (4.60–6.28)
**Usual source of health care**					
No usual place for health care	18 (2.2)	834	111 (16.2)	684	7.08 (4.36–11.48)
Clinic or health care center	59 (6.1)	970	426 (37.0)	1,153	5.68 (4.36–7.38)
Doctor’s office or HMO	136 (7.2)	1,881	850 (41.7)	2,039	5.34 (4.41–6.46)
Other place for health care	2 (2.2)	92	30 (22.9)	131	9.69 (2.38–39.38)
**Participated in a behavioral Intervention within the past 12 months**					
No	118 (4.2)	2,842	858 (30.7)	2,797	6.64 (5.38–8.19)
Yes	98 (10.0)	979	565 (45.1)	1,253	4.03 (3.31–4.90)
**Tested for HIV within the past 12 months**					
No	3 (0.4)	838	19 (3.2)	602	8.33 (2.46–28.24)
Yes	213 (7.2)	2,976	1,406 (40.8)	3,444	5.26 (4.51–6.12)
**Region^¶^**					
Midwest	27 (7.6)	353	117 (32.7)	358	3.91 (2.35–6.52)
Northeast	46 (5.8)	793	293 (36.9)	794	5.78 (4.21–7.95)
South	69 (5.1)	1,350	409 (29.6)	1,383	5.44 (4.18–7.08)
U.S. territories	2 (0.9)	228	7 (5.7)	123	5.08 (1.19–21.74)
West	72 (6.6)	1,097	599 (43.0)	1,394	6.36 (4.87–8.30)
**Urban area**					
Atlanta, GA	12 (6.3)	192	56 (28.1)	199	4.29 (2.08–8.84)
Baltimore, MD	8 (5.1)	157	20 (18.5)	108	3.39 (1.53–7.55)
Boston, MA	11 (7.6)	145	105 (50.0)	210	6.33 (3.16–12.65)
Chicago, IL	23 (11.7)	197	93 (47.2)	197	3.79 (2.22–6.47)
Dallas, TX	4 (2.2)	178	63 (25.1)	251	11.12 (3.52–35.16)
Denver, CO	4 (1.9)	210	92 (31.9)	288	15.71 (5.97–41.30)
Detroit, MI	4 (2.6)	156	24 (14.9)	161	5.49 (2.05–14.66)
Houston, TX	9 (4.8)	187	60 (24.5)	245	4.66 (2.48–8.75)
Los Angeles, CA	11 (4.2)	259	109 (36.9)	295	9.13 (4.97–16.78)
Miami, FL	5 (2.4)	211	30 (17.6)	170	7.75 (3.26–18.41)
Nassau and Suffolk counties, NY	3 (1.9)	159	15 (18.5)	81	9.81 (3.03–31.79)
New Orleans, LA	5 (2.8)	181	65 (39.4)	165	12.99 (5.55–30.43)
New York City, NY	8 (5.1)	156	101 (40.7)	248	6.88 (3.61–13.10)
Newark, NJ	1 (1.1)	88	13 (24.1)	54	21.15 (2.97–150.41)
Philadelphia, PA	23 (9.4)	245	59 (29.4)	201	3.20 (2.03–5.04)
San Diego, CA	12 (5.5)	218	120 (40.8)	294	7.34 (4.11–13.13)
San Francisco, CA	26 (14.9)	174	164 (61.2)	268	3.93 (2.55–6.04)
San Juan, PR	2 (0.9)	228	7 (5.7)	123	5.08 (1.19–21.74)
Seattle, WA	19 (8.1)	236	114 (45.8)	249	5.44 (3.34–8.85)
Washington, DC	26 (10.7)	244	115 (46.9)	245	4.54 (3.08–6.70)

**Abbreviations:** CI = confidence interval; HMO = health maintenance organization.

* Men who were at risk for HIV infection and likely to meet clinical indications for HIV preexposure prophylaxis. This was defined as men who had a negative HIV test result at the time of the interview, did not report a previous HIV-positive test result, had either one male sex partner who was HIV-positive or multiple male sex partners in the past 12 months, and reported either condomless anal sex or a sexually transmitted bacterial infection in the past 12 months.

^†^ Models adjusted for income, health insurance, and region.

^§^ Includes American Indian, Alaskan Native, Asian, Native Hawaiian, Pacific Islander, or multiple races.

^¶^ *Midwest region* includes Chicago, IL and Detroit, MI. *Northeast region* includes Boston, MA; Nassau and Suffolk counties, NY; New York City, NY; Newark, NJ; and Philadelphia, PA. *South region* includes Atlanta, GA; Baltimore, MD; Dallas, TX; Houston, TX; Miami, FL; New Orleans, LA; and Washington, DC. *U.S. territories region* includes San Juan, PR. *West region* includes Denver, CO; Los Angeles, CA; San Diego, CA; San Francisco, CA; and Seattle, WA.

## Discussion

From 2014 to 2017, PrEP awareness among MSM in this analysis increased by 50%. More importantly, in 2017, >80% of MSM in all racial and ethnic groups and in 17 of the 20 urban areas were aware of PrEP. This finding is encouraging and suggests that efforts designed to increase PrEP awareness among populations at risk for HIV infection are having a positive impact. These efforts have included media and social marketing campaigns (e.g., Act Against AIDS^¶¶^). In addition, national HIV prevention goals were updated in 2015 to expand efforts to prevent HIV infection using a combination of effective, evidence-based approaches among populations with the highest prevalences of HIV infection, including among black and Hispanic MSM (*5*). Thus, continued increases of awareness among MSM, especially among black and Hispanic MSM, are expected.

Although PrEP use by MSM in this analysis increased approximately 500% from 2014 to 2017, only approximately one in three men at risk for HIV infection reported using PrEP. Models examining the impact of PrEP use on incidence predict that the use of PrEP by 30%–40% of MSM with PrEP indications in a community could result in approximately one third of new HIV infections being averted over a 10-year period, with a greater predicted impact if coverage is increased (*6*). The reported increase in PrEP use among MSM is promising, but higher coverage is needed to reduce incidence of new infections by 90% within the 10 years of the Ending the HIV Epidemic initiative.

The overall impact and efficiency of PrEP at averting new infections is greater in communities with a high prevalence of HIV (*7*,*8*). Therefore, efforts focused on increasing PrEP use among black and Hispanic MSM, who have a higher prevalence of HIV infection (*3*), might substantially reduce the incidence of HIV infections. The large percentage increases in PrEP use among black and Hispanic MSM in this analysis are promising, but PrEP use in these groups remains low; continued efforts will be needed to meet the goals of the Ending the HIV Epidemic initiative. Because of the structural barriers associated with race that influence access to quality health care (*9*), demonstration projects for the Targeted Highly-Effective Interventions to Reverse the HIV Epidemic (THRIVE) program*** are underway in seven U.S. cities. These projects establish community collaboratives that provide comprehensive HIV prevention and care services for black and Hispanic MSM. Lessons learned from these efforts might help further inform how best to increase PrEP use among these populations.

Some health care providers might be missing opportunities to provide PrEP to patients who would benefit from its use. MSM included in this analysis reported behaviors that put them at substantial risk for HIV infection, yet only 39% of those who saw a health care provider in the past 12 months reported using PrEP. CDC’s HIV PrEP clinical practice guideline offers comprehensive information to providers for prescribing and managing PrEP and recommends that health care providers take routine sexual histories of all their patients (*2*). However, some providers only take a sexual history if it is related to the patient’s complaint and ask nonspecific questions about sex (*10*). To increase PrEP use, health care providers might need training and resources to ensure they know how to assess their patients for indications for PrEP and are confident discussing PrEP medication. As part of CDC’s Act Against AIDS communication campaign, the Prescribe HIV Prevention program offers an online toolkit to help health care providers use PrEP to prevent new HIV infections among patients at high risk. This toolkit includes resources such as answers to frequently asked questions about PrEP medication and its related clinical care, campaign posters to help raise PrEP awareness, patient materials, a tool to aid health care providers in discussing sexual histories with their patients, and continuing medical education courses on PrEP. To fulfill their critical role in reducing new HIV infections in the United States, health care providers will need to routinely test patients for HIV, link those with HIV infection to care, and discuss HIV prevention options (e.g., condoms and PrEP) with those who are not infected.

The findings in this report are subject to at least six limitations. First, NHBS data do not correspond directly with the criteria for PrEP indication in the clinical guidelines. NHBS uses a 12-month period for assessing risk behaviors versus a 6-month period specified in the clinical guidelines. Second, this analysis used having two or more sex partners in the past year as a proxy for a nonmonogamous relationship, but these partnerships might not have overlapped in time. Thus, the analysis might include some men without indications for PrEP use. Their inclusion in the denominator might underestimate the percentage of men in NHBS using PrEP. Third, different questions were used to assess PrEP awareness and use in 2014 and 2017. The measure of PrEP use in 2017 was more specific than that in 2014, so estimates of PrEP use increases are potentially underestimated. Fourth, NHBS is not nationally representative and might not be generalizable to all cities, nonurban areas, or MSM. Fifth, because data were not weighted to account for the complex sampling methods used to recruit MSM, estimates might be biased by over- or underestimating subgroups of the population. Finally, data on self-reported behaviors might be subject to recall and social desirability biases. Although the impact of recall bias on the analysis is unknown, social desirability bias might lead to overreporting PrEP awareness and use.

HIV PrEP awareness and use is increasing in the United States among MSM who are at risk for acquiring HIV, but higher coverage is needed, especially among black and Hispanic MSM, to end the HIV epidemic in the United States by 2030. By routinely testing their patients for HIV, assessing HIV-negative patients for risk behaviors, and prescribing PrEP as needed, health care providers can play a critical role in this effort.

**Summary**

**What is already known about this topic?**

Men who have sex with men (MSM) can reduce their risk for human immunodeficiency virus (HIV) infection by using preexposure prophylaxis (PrEP) consistently. Increasing PrEP use is a principal strategy of the Ending the HIV Epidemic initiative.

**What is added by this report?**

From 2014 to 2017, PrEP awareness among MSM in 20 urban areas increased from 60% to 90%, and PrEP use increased from 6% to 35%. PrEP use increased in almost all demographic subgroups but remains lower among black and Hispanic MSM.

**What are the implications for public health practice?**

By routinely testing patients for HIV, assessing HIV-negative patients for risk behaviors, and prescribing PrEP as needed, health care providers can play a critical role in ending the HIV epidemic.

## Footnotes

* https://www.hiv.gov/federal-response/ending-the-hiv-epidemic/overview?s_cid=ht_endinghivinternet0002.

† https://aidsvu.org/ending-the-epidemic/.

§ https://www.cdc.gov/actagainstaids/campaigns/prescribe-hiv-prevention/index.html.

¶ The number of U.S. urban areas collecting data differed in 2014 and 2017. The following 20 urban areas collected data both years: Atlanta, Georgia; Baltimore, Maryland; Boston, Massachusetts; Chicago, Illinois; Dallas, Texas; Denver, Colorado; Detroit, Michigan; Houston, Texas; Los Angeles, California; Miami, Florida; Nassau and Suffolk counties, New York; New Orleans, Louisiana; New York City, New York; Newark, New Jersey; Philadelphia, Pennsylvania; San Diego, California; San Francisco, California; San Juan, Puerto Rico; Seattle, Washington; and Washington, DC. The following three urban areas that collected data in 2017 were not included in this analysis: Memphis, Tennessee; Norfolk, Virginia; and Portland, Oregon.

** Men who were born male and identified as male, reported having ever had oral or anal sex with another man, resided in the interview city, were aged ≥18 years, and could complete the interview in English or Spanish.

†† The incentive format (cash or gift card) and amount varied by city according to formative assessment and local policy. A typical format included $25 for completing the interview and $25 for providing a specimen for HIV testing.

§§ NHBS data do not correspond directly with the criteria for PrEP indication in the clinical guidelines. The guidelines recommend that men use PrEP if they are without acute or established HIV infection, have had sex with a nonmonogamous male partner who has not recently tested HIV-negative, and have had at least one of the following: any anal sex without a condom in the past 6 months or a bacterial STI (i.e., syphilis, gonorrhea, or chlamydia) diagnosed or reported in the past 6 months. NHBS data flag persons who are likely indicated for PrEP use because of behavior from a longer period (12 months versus 6 months) and use multiple sex partners as a proxy for a nonmonogamous partner.

¶¶ https://www.cdc.gov/actagainstaids/index.html.

*** https://www.cdc.gov/hiv/research/thrive/about.html.
